# Môle hydatiforme partielle invasive et métastatique: à propos d'un cas

**DOI:** 10.11604/pamj.2014.19.175.5487

**Published:** 2014-10-20

**Authors:** Ikram Lazrak, Hakimi Ihssane, Moulay Abdellah Babahabib, Jaouad Kouach, Mohamed Reda El Ochi, Moulay Driss Moussaoui, Mohamed Dehayni

**Affiliations:** 1 Service de Gynécologie Obstétrique de l‘Hôpital Militaire Med V de Rabat, Rabat, Maroc; 2 Service d'Anatomo-Pathologie de l‘Hôpital Militaire Med V de Rabat, Rabat, Maroc

**Keywords:** Môle hydatiforme partielle, môle invasive, métastases pulmonaires, métastases vaginales, chimiothérapie, partial hydatiform mole, invasive mole, pulmonary metastases, vaginal metastases, chimiotherapy

## Abstract

Depuis plusieurs années, la môle hydatiforme partielle(MHP) a été considérée comme une entité bénigne qui ne nécessite pas une surveillance stricte comme celle de la môle complète(MC), mais l'apparition de quelques cas sporadiques de transformation de la môle partielle en maladie trophoblastique persistante que ça soit une môle invasive ou choriocarcinome ou voire même une tumeur du site d'implantation placentaire; a remis en question cette stratégie. A travers une observation d'une môle partielle invasive métastatique, et à travers une revue de la littérature, on a essayé d'appuyer cette conduite qui considère la môle partielle comme une pathologie potentiellement grave nécessitant une prise en charge adéquate et une surveillance assez rigoureuse.

## Introduction

Historiquement, la môle partielle, embryonnée, (du latin *moles*: masse) désignait un produit de conception avec des villosités molaires entourant une cavité amniotique pourvue d'un embryon. Elle était considérée comme une variante de la môle hydatiforme complète. Actuellement, la môle partielle, embryonnée, est séparée nosologiquement de la môle complète hydatiforme puisque sa nature est presque toujours triploïde alors que la mole hydatiforme complète est souvent diploïde et généralement d'origine paternelle. La fréquence des môles partielles est estimée entre 10 et 20% des produits d'avortements spontanés [1]. Elles passent inaperçues en l'absence d'examen histologique ou cytogénétique systématique. Le risque de môle partielle est différent de celui de la môle complète [2]. Il est accru en cas de rythmes menstruels irréguliers et de contraception orale depuis plus de 4 ans [3]. En revanche, le risque n'est majoré ni par l’élévation de l’âge maternel [4], ni par le régime alimentaire. L’âge d'apparition de la môle partielle correspond à celui de la période de fertilité de la femme. L’évolution des môles partielles est bénigne dans 97% des cas et se fait vers une maladie trophoblastique persistante dans 0,5 à 3% des cas. Des môles partielles invasives ainsi que des choriocarcinome après une mole partielle ont été rapportées dans moins de 3% des cas [5–8]. Dans cette observation, nous rapportons le cas d'une môle hydatiforme partielle avec composante invasive et des métastases à distance chez une patiente de 42 ans.

## Patient et observation

**Figure 1:**
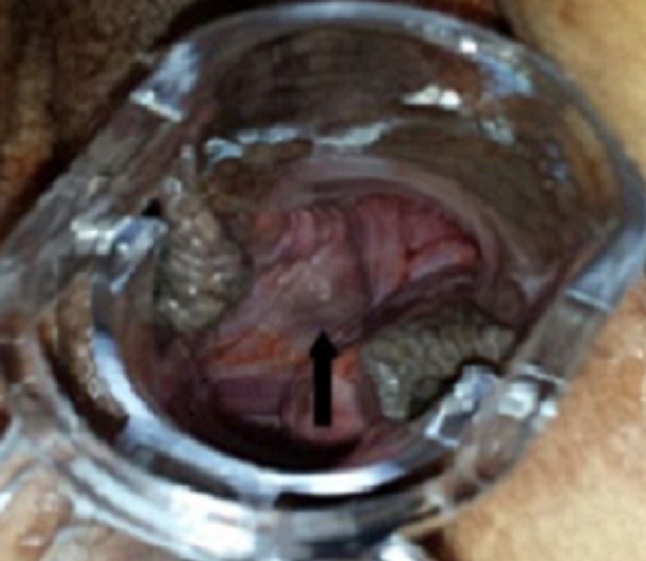
Lésion bleuâtre de 1cm au niveau de la paroi antérolatérale droite du vagin à la jonction des 2/3 sup en rapport avec une localisation secondaire de la môle partielle

**Figure 2:**
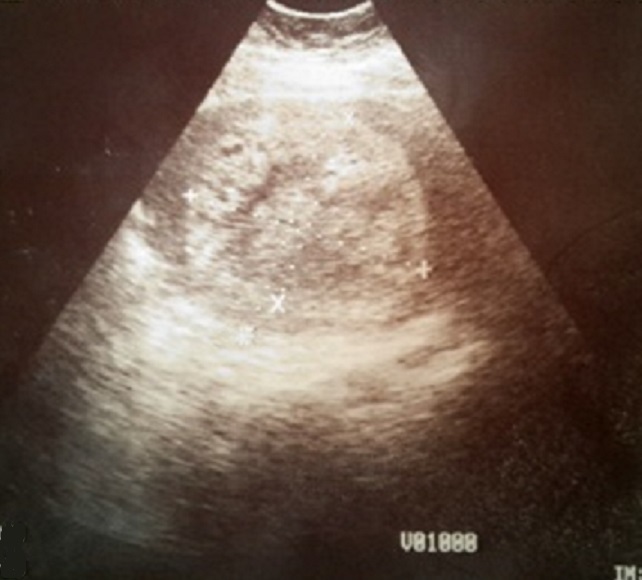
Échographie pelvienne: aspect échographique de la môle partielle hyperéchogéne hétérogène intra-cavitaire

**Figure 3:**
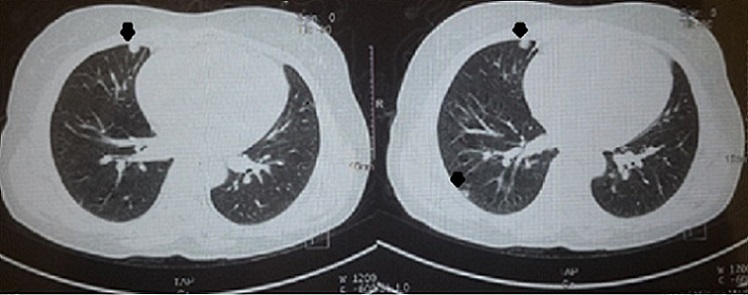
TDM thoracique: Images nodulaires intraparenchymateuses pulmonaire en rapport d'une localisation secondaire de la môle partielle

**Figure 4:**
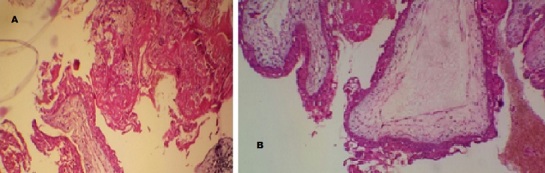
Môle incomplète. Villosité placentaire bordés par une hyperplasie focale atypique des cellules trophoblastiques(a), en certains endroits, les cellules du cyto-trophoblaste présentent une dystrophie bulleuse(b). (HES ×100)

Il s'agit de madame D.M âgée de 42 ans de groupe sanguin B Rh+, mère de deux enfants suivie pour gastrite chronique, qui a présenté des métrorragies sur une aménorrhée de 10 SA prises par la patiente pour des troubles de cycles en rapport avec la péri-ménopause, ainsi que des vomissements que la patiente avait rattaché à sa gastrite et elle n'a consulté qu'1 mois après échec des traitements hémostatiques et antiémétiques pris en ambulatoire, l'examen a trouvé une patiente en bon état général, stable sur le plan hémodynamique et respiratoire, bien orientée dans le temps et l'espace, avec a l'examen gynécologique: une hauteur utérine supérieur à l’âge gestationnel théorique, au speculum un col violacé avec un saignement minime provenant de l'endocol et une lésion bleuâtre régulière lisse indurée de 1cm au niveau de la paroi antérolatérale droite du vagin à la jonction des 2/3 sup (Figure 1); au toucher vaginal, l'utérus faisait 20 SA (> AGT) mou, sans perception de masse en latéro-pelvien. Un dosage de beta HCG a été fait revenant à 364491 mUI/mL, l’échographie pelvienne a montré une image intracavitaire hétérogène faisant 11cmde grand axe, infiltrant le myometre ainsi que des zone d'hypervascularisation focale au niveau de la paroi utérine antérieure sans visualisation de kystes lutéiniques en latéro-utérin (Figure 2). La patiente a bénéficié d'une aspiration prudente écho-guidée sous couverture antibiotiques et utéro-toniques et le produit d'aspiration a été adressé à l’étude histologique. Devant la présence de la lésion vaginale et les signes d'invasion myometriale on a réalisé d'emblée un bilan d'extension à savoir une IRM cérébrale et un scanner thoraco-abdomino-pelvien qui ont révélés de multiples lésions nodulaires intraparenchymateuses diffuses aux deux champs pulmonaires en rapport avec une localisation secondaire (Figure 3). L’étude histologique est revenue en faveur d'une môle hydatiforme incomplète avec présence d'une hyperplasie focale (Figure 4, a,b). Selon le score pronostique de FIGO 2000 notre patiente a été considéré a haut risque (score = 10) et elle a reçu une polychimiothérapie à type d'EMA-CO avec une bonne tolérance et bonne évolution sur un recul de 6 mois.

## Discussion

La môle hydatiforme partielle résulte d'un mélange de vésicules môlaires et de villosités placentaires normales avec un tissu embryonnaire reconnaissable, elle est presque toujours triploïde. Le jeu supplémentaire de chromosomes est d'origine paternelle (diandrie) dans 85% des cas, ou d'origine maternelle (digynie) dans 15% des cas. La triploïdie paternelle vient de la fertilisation d'un ovule normal par un spermatozoïde anormal diploïde (deux jeux chromosomiques) ou par deux spermatozoïdes haploïdes normaux (un jeu de chromosomes). En revanche, la triploïdie maternelle est le résultat de la fertilisation d'un ovule anormal diploïde par un spermatozoïde normal haploïde. Le spermatozoïde et l'ovule anormalement diploïdes résulteraient d'une erreur de la première ou parfois de la deuxième division méiotique. Lorsque la triploïdie est d'origine paternelle, le placenta possède le phénotype particulier de môle partielle tandis que lorsque la triploïdie est d'origine maternelle, le placenta paraît morphologiquement normal (15% des triploïdies).

Après avoir cru que l'origine paternelle de la triploïdie prédominait, l’étude de l'empreinte génomique montrerait, au contraire, la dominance de l'origine maternelle [9]. Elle s'expliquerait par une meilleure survie des triploïdies digyniques.). Les môles partielles se singularisent par un arrêt du développement à différentes phases de la grossesse, allant des premières semaines jusqu’à la 24^e^ semaine et même au-delà, c'est-à-dire de l’œuf microscopique à la môle embryonnée [10]. Il n'y a pas de différences fondamentales entre la môle partielle découverte au premier trimestre, celle du deuxième trimestre et celle du troisième trimestre avec enfant vivant, porteur d'une triploïdie en «mosaïque » [11]. Lorsque l'embryon ou le fœtus sont présents, ils présentent un retard de croissance intra-utérin et, assez souvent, des malformations congénitales multiples [12]. Les signes cliniques de la grande majorité des môles partielles sont semblables à ceux des autres avortements spontanés.

L’échographie est nettement moins performante en cas de MP et la distinction échographique entre môle partielle et complète peut être difficile d'où l'examen anatomopathologique systématique de tout produit d'expulsion, elle peut révéler un œuf clair et, parfois, des microvésicules difficiles à distinguer de la dégénérescence hydropique focale des villosités d’œufs morts. Mais lorsque l’échographie montre des kystes dispersés dans le placenta et que le diamètre du sac gestationnel est augmenté, le diagnostic prédictif d'une môle partielle est estimé à 90% des cas [13], comme dans notre observation où on avait suspecter le diagnostic de la mole hydatiforme sur une image intracavitaire hétérogène, infiltrant le myomètre ainsi que des zone d'hypervascularisation focale au niveau de la paroi utérine antérieure sans visualisation de kystes lutéiniques en latéro-utérin, confirmé par l'examen histologique.

Le taux d'hCG est dans les limites de la normale, à l'exception de 2 à 3% des cas. Seules les môles partielles avec hyperplasie trophoblastique peuvent présenter des particularités cliniques, ainsi que des complications identiques à celles de la môle complète et peuvent s'accompagner d'un taux d'hCG supérieur à 100 000 mUI/mL. La fréquence réelle des hyperplasies trophoblastiques parmi les triploïdies et les tétraploïdies, se compliquant d'un taux élevé de bêta-hCG, n'est pas connue [14]. Elle est estimée à 0,1% des produits d'avortements (compte tenu du fait que les triploïdies et les tétraploïdies représentent respectivement 20 et 5% des produits d'avortements spontanés, et qu'elles se compliquent d'hyperplasie trophoblastique dans 3% des cas) [14]. Par ailleurs, des études rétrospectives de môles complètes montrent que 10 à 15% d'entre elles correspondent à des môles partielles. Le diagnostic positif de la mole partielle est porté à l'examen macroscopique et surtout histologique du matériel expulsé, aspiré ou cureté. Histologiquement, le diagnostic de môle partielle (syndrome triploïde) est porté grâce aux signes suivants associés ou isolés: alternance de villosités normales et anormales (100% des cas), contours villositaires en « fjord » (90%), citernes intrachoriales (75%) et méandres (3%), invaginations intrachoriales du trophoblaste aboutissant à des kystes trophoblastiques intrachoriaux (70%), dystrophies bulleuses trophoblastiques en excès. La citerne constitue le signe le plus objectif mais elle se trouve aussi dans la môle complète. L'embryon n'est pas toujours apparent mais ses traces peuvent persister: cordon ombilical résiduel, revêtement amniotique et/ou globules rouges nucléés dans la circulation sanguine f'tale des villosités.

L’évolution des môles partielles est bénigne dans 97% et le risque de dégénérescence une maladie trophoblastique persistante et invasive est faible par rapport à la MC et varie selon les études entre 0,5 et 3%. D'après une revue de la littérature [5, 7, 8] seules les môles partielles avec hyperplasie trophoblastique doivent être suivies par dosage des bêta-hCG comme pour une môle complète. Cette surveillance aussi rigoureuse des bêta-hCG a longuement été débattue. Certains arguaient que seules les MC, et non les MP, pouvaient se transformer en tumeur trophoblastique persistante voire un choriocarcinome alors que Bagshawe et al., dans une série ancienne, retrouvaient que 15% des MC et 0,5% des MP pouvaient développer une lésion maligne nécessitant une chimiothérapie [7]. En fait, c'est Bagshawe et al. qui ont suggéré que les MP pouvaient devenir des choriocarcinomes même si la preuve formelle manquait. À londre, à Charing Cross Hospital, trois mille patientes ayant présenté une MP entre 1980 et 2000 ont été analysées. Quinze de ces patientes ont par la suite développé une TT nécessitant une chimiothérapie [15]. La transformation possible d'une môle partielle en choriocarcinome a été démontrée par M. Seckl [8] et elle a été estimée de l'ordre de 0,5% après une môle hydatiforme partielle. En chine XI ZHOU et al. ont rapporté le cas d'une transformation invasive d'une môle partielle avec grossesse gémellaire suite à une fécondation in vitro [5] et dans leur étude ils ont estimés ce risque à environ 3%.

En clair, si les MP peuvent se transformer en tumeur trophoblastique persistante, il est essentiel de les détecter précocement pour éviter des complications très graves mettant en jeu le pronostic vital chez des femmes en âge de procréer. Le diagnostic de môle invasive est porté lorsque des villosités môlaires sont suspectées soit dans le myomètre, sur une hyperéchogénicité par examen échographique transvaginal ou en raison d'une hypervascularisation focale au doppler couleur, c’était d'ailleurs le cas de nôtre patiente, soit en dehors de l'utérus; et/ou lorsque le taux des hCG est anormalement persistant ou en réascension après une grossesse môlaire, sans môle résiduelle dans la cavité utérine confirmée par échographie endovaginale. Le siège des villosités greffées est préférentiellement le myomètre, parfois la paroi vaginale, plus rarement le péritoine et les poumons. Leur nombre est limité et leur existence est temporaire, excédant rarement 4 mois. Mais ces greffes sont graves en raison des hémorragies importantes qu'elles peuvent déclencher et, surtout jadis, par leurs complications infectieuses, jugulées actuellement par les antibiotiques. Les complications hémorragiques pouvant être mortelles, elles contre-indiquent toute biopsie, même vaginale. Or, le diagnostic de certitude d'une môle invasive ne peut être porté qu’à l'histologie et l'hystérectomie est seulement indiquée en période périménopausique ou lorsque la patiente refuse le suivi régulier [15].

Les indications d'un bilan d'extension lors d'une môle hydatiforme partielle ou d'un syndrome triploïde avec hyperplasie trophoblastique sont dictées par le taux anormal des bêta-hCG, croissant ou d'emblée, élevé, courbe s'infléchissant mal ou stagnant en plateau. Le bilan comporte: l'examen clinique afin d’éliminer, entre autres, une hyperthyroïdie, une prééclampsie; l’échographie endovaginale à la recherche d'un envahissement du myomètre; la radiographie du thorax au moment du diagnostic et, éventuellement, 4 semaines après; un bilan électrolytique et un bilan hématologique à la recherche d'une anémie et d'une leucopénie avant une éventuelle chimiothérapie.

Le traitement consiste à assurer l’évacuation complète de la cavité utérine par une aspiration endo-utérine écho-guidée (le curetage est à éviter car il tend à favoriser la pénétration de villosités dans les vaisseaux béants du myomètre) avec prévention des hémorragies utérines par administration concomitante des utero-toniques [15]. Si l'envahissement est localisé à l'utérus et si la patiente demande une stérilisation chirurgicale, presque toujours en période périménopausique, ou lorsque le suivi paraît incertain, une hystérectomie totale peut être pratiquée en préservant les ovaires, même s'ils portent de nombreux follicules kystiques. L'hystérectomie doit être suivie de la surveillance du taux d'hCG car des métastases peuvent préexister. Si l'hystérectomie n'est pas pratiquée, toutes les malades avec une maladie trophoblastique persistante, avec ou sans métastases démontrées, doivent être soumises à une chimiothérapie selon leurs risques établie par Classification pronostique des tumeurs trophoblastiques gestationnelles d'après la Fédération internationale des gynécologues et obstétriciens (FIGO) 2000. Seules les môles hydatiformes et quelques rares môles partielles (syndromes triploïdes) ayant un taux de bêta-hCG restant en plateau ou ascendant, sans métastases, sont traitées par une monochimiothérapie utilisant le méthotrexate, un agoniste de l'acide folique, ou l'actinomycine D. Lorsque le taux de bêta-hCG est normal pendant 3 semaines consécutives, le contrôle sérique devient mensuel pendant 12 mois, au cours desquels une contraception stricte orale est instaurée. En cas de métastases ou d'autres facteurs élevant le risque de la môle invasive, il convient d'associer le méthotrexate et l'actinomycine D, d'administer une triple chimiothérapie MAC (méthotrexate, actinomycine, cyclophosphamide) ou l'association étoposide-cisplatine ou alors d'utiliser, comme chez notre patiente, Etoposide mathotrexate Actinomycine, cyclophosphamide, vincristine (EMA-CO) avec des résultats satisfaisant et un bon recul de 6 mois. La polychimiothérapie paraît réduire de 40 à 11% les môles hydatiformes à haut risque [15]. L’évolution est en générale favorable après une chimiothérapie, avec une nette diminution des taux de récidive.

## Conclusion

La môle hydatiforme partielle est une pathologie bénigne qui peut parfois avoir des transformations malignes pouvant mettre en jeu le pronostic obstétrical voire vital de jeunes femmes en âge de procréer, d'où la nécessité d'une prise en charge adéquate et une surveillance assez rigoureuse.
